# In the Children's Ward

**Published:** 1887-03-19

**Authors:** Walter J. Eccott


### In the Children's Ward.

By Walter J. Eccott.

### Chapter III.

Mrs. Inman, it may be surmised, was not long after the earliest possible minute next day in making her appearance in Sister Mercy's ward at St. Elizabeth's. The surgeon had ordered Elsie a sleeping-draught the night before, in view of the feverish restlessness sure to accompany the first night after the fracture of a limb; so that the little patient had obtained some rest. She was sitting up in bed when her mother came in, looking about her with vivid interest at the unaccustomed scene. Several other mothers and friends had come, like Mrs. Inman, and the ward presented an appearance of cheerful activity. The prattle of children resounded from the various beds, save where there were no visitors; and there, little eyes looked wistfully at those who were more blessed. The Sister left Mrs. Inman, after a minute or two, alone with her child. Elsie had recovered from the shock of yesterday sufficiently to find her tongue. In her childish way she talked volubly; and Dolly, who had not been forgotten, came in for a great share of attention. Elsie seemed rather proud of her bandaged leg than otherwise, and wanted to show it.

"Muvver, 'ook at Elsie's 'eg," she reiterated, and she seemed quite satisfied when mother told her it would soon get well. She called mother's attention to "'ittle 'ed 'idin' 'ood" opposite. There was one at home, and she thought it had been brought here. "Dadda bing 'ed 'idin' 'ood up 'ere?" she inquired. And when mother said "No, dear!" she seemed puzzled, and then put on a serious look, as if she was considering possibilities. Then she suggested "'ittle 'ed 'idin' 'ood tum herself?" to which mother assented.

Presently, as Elsie became satisfied with her mother being there, and thinking she had come to stay, and that there was no immediate occasion for any great display of affection or attention, she became more and more absorbed with Dolly. Then Sister Mercy came up, and began talking to Mrs. Inman. The latter was nothing loth, and presently Sister Mercy, who was a good listener, heard other scraps of private history, as she had heard many before. Very soon Mrs. Inman told her how George got thrown out of work, and found a sympathiser for their case in her listener. She even told where he had worked, with all that particularisation of detail usual with women of the artisan class, and in all confidence not doubting that her listener must feel equal interest with herself. She was not far wrong in this instance. What made Sister Mercy so successful with her children-patients, so loved by them and their parents, was her inexhaustible patience in listening to, her ready grasp of, and memory for these family associations, which went far to form strong links of sympathy with them. Mrs. Inman told how the news of her husband's misfortune came to her just before that of the greater temporary trouble of Elsie's accident. Again she received a comforting assurance that it was not serious, that no after-effects need be feared. Then Mrs. Inman descanted eloquently on the brightness and cleverness of little Elsie, and Elsie, hearing her name mentioned, frequently chimed in. The all too brief hour came to a close, and once again, with a lighter heart, the mother took leave of her greatest treasure.

So days came and went for nearly a week. Mrs. Inman came regularly. Elsie grew better and better. She was ready to talk of "Dotty Feece," as she called him, and "Sissy Mercy," in a way that was comical to hear; and she was a great favourite with both.

George had got no work yet. He was beginning to despair of any early prospect. He had tried so many shops. More than once he had been to visit Elsie. His intelligence and his fatherly fondness made Sister Mercy quite take to him. She was ready to stand his friend on the first occasion that should arise.

One day Doctor Fierce brought a gentleman into the ward, a tall, commanding man with a fine intellectual face. When an official of St. Elizabeth's showed a visitor round, it was done in no merely official way. Everything was explained, everything at all likely to interest was shown courteously and willingly, for the officials at St. Elizabeth's had their hearts in their work. And who knew what might result for their darling hospital from a stray crumb of courtesy? Many a cheque might flow in, which otherwise would not. Doctor Fierce introduced the visitor to Sister Mercy, and the visitor at once commented on the artistic appearance of the ward, and seemed greatly interested in the tale of the self-devotion of the lady artist who had so adorned it. Then the Doctor pointed out his various little charges, and as he was passing the bed opposite Red Riding Hood, a little voice called:

"Dotty Feece! Dotty Feece!"

"Charles!" said Doctor Fierce, "here's a pretty little girl I've got to show you," turning in that direction, and both stood at Elsie's bedside.

"Well, Elsie?" said the Doctor kindly.

"Want to 'ook at Elsie's 'eg?" said the patient, not a whit abashed as the Doctor turned back the clothes. And when she tried to lift up the bandaged leg in her tiny hands, the visitor could not help smiling.

"I say, Elsie! won't you sing me a song?" said the Doctor. "Here, Sister! doesn't Elsie sing?"

"Yes, sometimes," said Sister Mercy. "Sing 'Three blind mice,' there's a dear little Elsie!"

Miss Elsie started right away, with her blue eyes staring straight in front of her, and a most solemn expression:

> "Fee bind mice! Fee bind mice! Fee bind mice!
> See how vey wun," etc.

Right through the verse she sang in her comical way, and got up to the high notes at the end in a most ludicrously business-like style, which made Sister, Doctor, and visitor laugh heartily. At this outburst Elsie stared, and then said, in an inconsequential way, "Gemman kiss Dolly!" and held up her precious charge. The visitor performed the operation to Elsie's satisfaction evidently.

"Who is that child, Fierce?" said the visitor. "I feel interested."

"Perhaps you can tell us, Sister?" said the Doctor, turning to Sister Mercy.

"She's the only child of a George Inman, a gilder, who worked until recently at a place in Cavenmore Street, a large decorator's. Cartman, I think the name was."

"Or Cartland?" suggested the visitor.

"Yes, Cartland it was," said the Sister. "He was discharged the other day,—a very intelligent, superior man, I should say, from what I've seen of him. He's been trying everywhere to get work, but hasn't succeeded so far. His wife seems in great concern about it. They're both young people, and it seems a great pity, just when this other trouble came upon them. I don't know whether you could use any interest for him?"

Just at this point a little boy, who was well enough not to need to pass much of his time in bed, came up and pulled Sister Mercy by the dress, saying:

"Sister! can you please give me a piece of string?" and Sister Mercy went off to find the required article for the child. When she returned she heard the visitor saying:

"Inman? Inman? I fancy I've heard the name. I believe we had a man of that name in our shops. Thank you very much, Sister, for your information. If I hear anything I'll let you know. Trust me, this isn't any off-hand promise, made to be dismissed from my thoughts in a few minutes. I'm quite taken with your patient there. Good-day to you. I'll see what I can do." And he passed out with Doctor Fierce, remarking, as he did so, "How well your charges look!" and the Doctor, smiling, replied, "Don't blame me. It's all Sister Mercy's doings!"

Not many days after, George Inman received a note to call at the firm, and Mr. Charles Cartland told him he wanted him to go down in charge of a long country job. He advised him to take his wife and child down too, giving him a liberal cheque to pay all expenses, and would not hear of any thanks. "Give them to little Elsie!" he said with a smile, mystifying George exceedingly. And about the same time the secretary at St. Elizabeth's received a cheque from the firm of Cartlands Bros., endowing a bed in "The Children's Ward."

